# Review of “*Echinococcus and Echinococcosis, Part A.*” edited by R. C. Andrew Thompson, Alan J. Lymbery and Peter Deplazes

**DOI:** 10.1186/s13071-017-2345-8

**Published:** 2017-09-04

**Authors:** Akira Ito

**Affiliations:** Department of Parasitology, Asahikawa Medical University, Midorigaoka Higashi 2-1-1-1, Asahikawa, 078-8510 Japan

## Abstract

Thompson RCA, Deplazes P, Lymbery AJ, Editors. *Echinococcus and Echinococcosis, Part A. Volume 95, Advances in Parasitology* 95. Academic Press; 2017. 525 pages, ISBN 978-0-12-8114711 (hardcover); 9780128114728 (eBook).

## Review

Echinococcosis is the most lethal chronic hepatic disease caused by accidental ingestion of eggs of the genus *Echinococcus.* The life-cycle is completed through a predator-prey interaction. The parasite matures in the small intestine of domesticated and/or wild carnivores. This book may be described as the second edition of “*Echinococcus and Hydatid Disease*” edited by RCA Thompson and AJ Lymbery, published in 1995.

Approximately two decades ago, only four species of *Echinococcus* were recognized as rigid independent species. Among them, *E. granulosus* and *E. multilocularis* are of public health importance, since they cause cystic echinococcosis (CE) (hepatic abscess) and alveolar echinococcosis (AE) (hepatic malignant tumor), respectively.

Recent molecular studies have revealed that *E. granulosus* (*sensu* *lato*) (*s.l*.) consists of five independent species: *E. granulosus* (*sensu stricto*) (*s.s*.), *E. equinus*, *E. ortleppi*, *E. canadensis* and *E. felidis*. Among these five species in *E. granulosus* (*s.l*.), *E. granulosus* (*s.s*.) (G1) and *E. canadensis* (G6/7) are the major pathogens for human CE. Furthermore, a new species, *E. shiquicus*, a sister species of *E. multilocularis*, was described from the Tibetan plateau, China in 2005, and *E. felidis* from African lions, a sister species of *E. granulosus* (*s.s*.) (G1), was resurrected as an independent species from *E. granulosus felidis* in Africa in 2008. Therefore, it is really timely for us to have an updated review book on these topics.

People involved in the control of echinococcosis are expected to do any surveillance based on molecular identification of the pathogens. In Africa, it is easy to expect that human cases with *E. felidis* should not be rare. In Asia, mainly in the Tibetan plateau, China, *E. shiquicus* may also be confirmed from humans.

The definitive hosts, either domesticated or wild carnivores, have been contaminating our living environment with eggs. Accidental human infection is mainly caused by human invasion into the wild animal territories, but also by wild animals such as red foxes which also often invade our living environment in big cities in developed countries.

This book with six chapters is an updated overview on the highly pathogenic parasites in the genus *Echinococcus*. The authors are well known experts in each topic. Chapter 1 by Eckert and Thompson is on “Historical aspects of Echinococcosis” and is highly informative. It mainly focuses on the pioneer researchers and their scientific contribution with their memorial photos. It is interesting to learn what the first senior author, Eckert, has done for the establishment of WHO informal working group on echinococcosis etc. over 60 years’ lifework. If it had included the two new species, *E. shiquicus* and *E. felidis*, or the most recent debate on *E. canadensis*, it would be perfect.

Chapters 2 and 3 by Thompson and Lymbery, respectively, from the same group are on “Biology and Systematics of *Echinococcus*” and “Phylogenetic Pattern, Evolutionary Processes and Species Delimitation in the Genus *Echinococcus*”, respectively. Although they recently stressed that *E. canadensis* should be divided into three species, i.e. *E. intermedius*, *E. borealis* and *E. canadensis* (see Lymbery et al. [1]), Chapter 2 keeps *E. intermedius* and *E. canadensis*, and Chapter 3 keeps *E. canadensis* (Yanagida et al. [2]). Chapter 4 by Brehm and Koziol on “*Echinococcus* - Host Interactions at Cellular and Molecular Levels” is a highlight of this book and highly informative. It gives an updated overview on the biochemistry and molecular biology of the parasites and host-parasite interactions.

From Chapters 1–4, I still have no clear idea on the mechanism as to how the laminated layer emerges in daughter cysts in *E. granulosus* (*s.l*.) through endogenous budding, and the reverse topology of laminated layer in mother *vs *daughter cyst is resolved. But the laminated layer might be differentiated from undifferentiated stem cells in the germinal layer in the mother cyst.

Chapter 5 by Romig et al. on “Ecology and Life Cycle Patterns of *Echinococcus* Species” is informative with excellent figures showing the life-cycle of each species.

Chapter 6 by Deplazes et al. on “Global Distribution of Alveolar and Cystic Echinococcosis” is focused on the distribution of *E. multilocularis* and *E. granulosus* (*s.l*.) and highly informative in general. It clearly indicates the historical evidence for the distribution of different genotypes of *E. multilocularis* and different species of *E. granulosus* (*s.l.*) on a global scale. It is easy to understand that *Echinococcus* parasites have been traveling the world. It is the time for field and laboratory parasitologists to join together using molecular tools.

It is easy to believe that the living environment of humans is becoming more complicated than we expected before reading this book. Is it possible for us to eradicate echinococcosis? Or should we be patient to live together with those pathogens, but challenge for sustainable education and practice for prevention of echinococcosis? If an innovative metacestocidal drug is developed, its benefit would be enormous!

I dedicate this book review to the late Professor the Lord EJL Soulsby who passed away on 12 May 2017.

## Abbreviations

AE: Alveolar echinococcosis
CE: Cystic echinococcosis
